# Decision-making about HPV vaccination in parents of boys and girls: A population-based survey in England and Wales

**DOI:** 10.1016/j.vaccine.2019.11.046

**Published:** 2020-01-29

**Authors:** Jo Waller, Alice Forster, Mairead Ryan, Rebecca Richards, Helen Bedford, Laura Marlow

**Affiliations:** a Cancer Prevention Group, School of Cancer & Pharmaceutical Sciences, King’s College London, Guy’s Hospital, Great Maze Pond, London SE1 9RT, UK; b Cancer Communication & Screening Group, Department of Behavioural Science and Health, UCL, Gower Street, London WC1E 6BT, UK; c Population, Policy and Practice Research and Teaching Department, UCL Great Ormond Street Institute of Child Health, 30 Guilford Street, London WC1N 1EH, UK

**Keywords:** HPV vaccine, Attitudes, Precaution adoption process model, Adolescent, Boys

## Abstract

**Background:**

School-based HPV vaccination in the UK will soon be extended to boys. Based on other countries’ experience, uptake may initially be lower in boys than girls. We assessed HPV vaccine attitudes and decision-making in parents of boys and girls, to explore sex differences and inform public health messages.

**Methods:**

We carried out a cross-sectional population-based survey using home-based interviews in spring 2019. Participants were adults in England and Wales, with a child in school years 5–7 (aged 9–12 and eligible for HPV vaccination within 3 years). Measures included awareness of HPV and the vaccine, demographic factors, previous vaccine refusal and (after exposure to brief information) whether participants would allow their child to have the HPV vaccine (decided to vaccinate; decided not to vaccinate; undecided). We also assessed vaccine attitudes. Data were weighted to adjust for non-response. Multinomial logistic regression was used to explore predictors of deciding to (or not to) vaccinate compared with being undecided.

**Results:**

Among 1049 parents (weighted n = 1156), 55% were aware of HPV and the girls’ vaccination programme, but only 23% had heard of plans to vaccinate boys. After information exposure, 62% said they would vaccinate their child, 10% would not, and 28% were undecided. Parents of girls were more willing to vaccinate than parents of boys (adjusted odds ratio: 1.80 (1.32–2.45)). Positive attitudes and HPV/vaccine awareness were significantly independently associated with deciding to vaccinate. Previous vaccine refusal for a child was the strongest predictor of not wanting the HPV vaccine.

**Conclusions:**

Our findings suggest a need for public health campaigns to raise awareness of plans to extend HPV vaccination to boys. Reassuringly only 10% of all parents were unwilling to vaccinate and our data suggest further information, including about safety and efficacy, may be important in supporting undecided parents to make the decision to vaccinate.

## Highlights

• Awareness of HPV is only just over 50% in parents whose children will soon be eligible for vaccination.
• More parents of girls than boys said they would vaccinate their child against HPV.
• Fathers, and parents of boys were more likely to be undecided about HPV vaccination.
• Potentially modifiable HPV vaccine attitudes were strong predictors of intention to vaccinate.

## 1 Background

Vaccination against high-risk types of human papillomavirus (HPV) is a highly effective public health intervention to reduce the incidence of cervical cancer. It is already having a dramatic impact on precancerous abnormalities in Scotland [1] and Australia [2]. It is also expected to have a significant impact on other HPV-related cancers including anogenital and head and neck cancers, as well as genital warts [3], [4], and has already led to a reduction in the prevalence of vaccine-type oral HPV infections in the United States [5]. Many countries across the world have implemented vaccination programmes aimed at protecting girls from cervical cancer [4]. Some also now recommend the vaccine for boys to increase herd protection, and to reduce the risk of HPV-related cancers in men, particularly oropharyngeal cancer, and anal cancer in men who have sex with men (MSM) [6]. The success of such programmes depends on high levels of coverage in the target populations.

In England the school-based HPV vaccination programme, which has targeted girls aged 12–13 since 2008, will be extended to include boys of the same age from September 2019 [7], [8], [9]. Similar announcements have been made in Scotland, Wales and Northern Ireland [10], [11], [12] bringing the UK into line with other countries offering gender-neutral HPV vaccination, including Australia, Austria, Bermuda, Brazil, Canada, Croatia, Germany, Israel, Italy, Lichtenstein, New Zealand, Serbia, and the USA [13].

The success of any vaccination programme relies on acceptability and high coverage in the relevant population. Vaccinations are generally well accepted but uncertainty and negative attitudes can have a direct impact on uptake. Understanding the target population’s willingness to have vaccinations can help anticipate important barriers or concerns to inform education campaigns, and can contribute to the success of a vaccination programme. Research on parents’ attitudes to HPV vaccination for boys is currently lacking in the UK. A survey of 186 parents of teenage boys in England (carried out in 2016–17) found low awareness of the HPV vaccine; however once provided with a brief description, 85% of parents thought it should be offered to boys [14]. Currently, there are no UK population-based data on parents’ awareness that the vaccine is soon to be offered to boys or the acceptability and likely uptake. Two recent reviews of HPV vaccine uptake and attitudes did not identify any UK-based studies, with the vast majority of work being carried out in North America [15], [16]). Uptake has consistently been found to be lower in boys than girls [15]. Possible explanations include unclear benefits for boys because of the policy focus on girls, and less likelihood of physicians recommending vaccinating boys [15]. There is an urgent need to understand attitudes in the UK, where school-based vaccine delivery means that physician recommendation is less relevant.

**Fig. 1:**
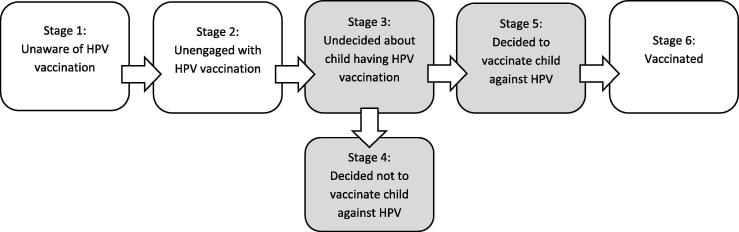
Precaution Adoption Process Model (PAPM) stages for HPV vaccination uptake (adapted from [18]). Note: The present study focuses on understanding Stages 3, 4 and 5 (shaded in grey).

A large representative survey of parents of boys and girls in Canada [17] used the Precaution Adoption Process Model (PAPM) [18]; see Fig. 1) to categorise parents’ stage of decision-making regarding HPV vaccination. The PAPM provides a useful framework to explore the stage parents are at in the process of taking up (or deciding not to take up) the HPV vaccine for their children. Parents can be considered to be in one of six stages: (1) unaware of the vaccine, (2) unengaged with the decision about whether to vaccinate, (3) undecided about vaccination, (4) decided not to vaccinate, (5) decided to vaccinate and (6) have vaccinated. Findings of the Canadian study revealed that parents of daughters (compared with sons), parents of older children, and parents who had received a healthcare provider recommendation were more likely to have moved further through the stages of the PAPM towards vaccine uptake. Additionally, the study explored the barriers to vaccination and reported that parents who were in the ‘decided not to vaccinate’ stage had significantly greater odds of reporting perceived vaccine harms, lack of confidence, risks and vaccine conspiracy beliefs.

The PAPM approach allows ‘vaccine hesitant’ parents to be categorised in a more nuanced way: those who are at the ‘unengaged’ or ‘undecided’ stages have been described as ‘flexible hesitant’, with beliefs that change over time, whereas the ‘decided not to vaccinate’ group are ‘rigidly hesitant’, with much more stable beliefs [19]. This distinction fits well with empirical evidence that highlights distinctions between children who are partially vaccinated and those who are fully vaccinated [20], [21].

The present study aimed to use the PAPM as a framework to gain a population-level understanding of the views of parents of boys and girls who will be offered HPV vaccination in England and Wales in the next three years. Building on the previous Canadian work [17], [19] we assessed: (1) awareness of HPV; (2) awareness of the HPV vaccination programme (for girls and boys); and (following information provision) (3) PAPM stage for HPV vaccine uptake (Stage 3, 4 and 5; see Fig. 1 [18]). We also explored attitudes to HPV vaccination.

## 2 Methods

### 2.1 Design

The study was a cross-sectional population-based survey. An outline protocol, including detailed research questions and analysis plan, was published on Open Science Framework prior to data collection (https://osf.io/3eycm/).

### 2.2 Participants and recruitment

The survey was carried out in England and Wales, where the vaccine is delivered to girls through schools in an identical way (Scotland was not included as the HPV vaccine is offered to slightly different age-groups making comparisons difficult; and Northern Ireland had not announced the roll-out of vaccination to boys at the time of study). To be eligible for the study, participants had to be living in England or Wales, aged 25 years or older, with a son or daughter in school year 5, 6 or 7 (aged 9–12 years and due to be offered the HPV vaccine in school year 8, at the age of 12–13 years, within the next three years). Participants who had more than one child in these school years, were asked to think of the oldest one when answering the questions (referred to as the ‘index child’).

### 2.3 Procedure

Data collection was carried out in February and March 2019 by Kantar TNS as part of their weekly omnibus survey. This means the survey was administered within a longer interview which covered other topics, commissioned by other organisations. Data were collected via home-based computer-assisted face-to-face interviews. Kantar TNS use stratified random location sampling to select areas across the UK which are visited by interviewers. At each location, quotas for age, sex, employment status and presence of children in the household are used to ensure the sample reflects the wider population. The study was approved by the UCL Research Ethics committee (ref: 3758/003).

### 2.4 Measures

The full questionnaire is available via Open Science Framework (https://osf.io/3eycm/).

**Fig. 2:**
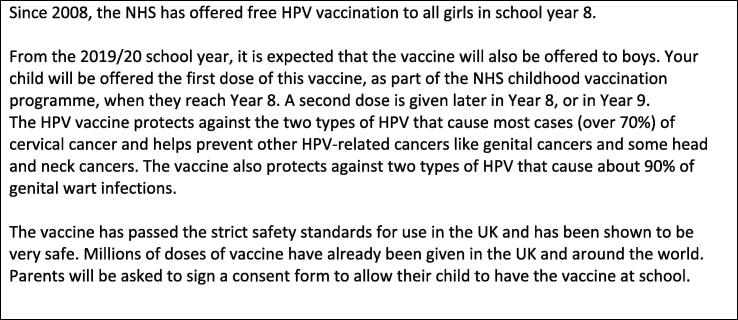
Information shown and read to participants during the survey.

PAPM stage for HPV vaccination: The primary outcome was PAPM stage for HPV vaccination for the index child, following exposure to brief information about the vaccine (see Fig. 2). Responses to the item ‘Having heard this information, do you think you will allow your son/daughter who is currently in year 5/6/7 to have the HPV vaccine when s/he is offered it in Year 8?’ (Yes/No/I’m not sure yet) allowed us to allocate participants to PAPM stage 3 (undecided about HPV vaccination), 4 (decided not to vaccinate the child) or 5 (decided to vaccinate).

Awareness of HPV and the vaccine: We measured previous awareness of HPV, awareness of the vaccination programme for girls and of the plan to extend this to boys using simple items (responses dichotomised into ‘yes’ vs. ‘no or unsure’) prior to exposure to the information in Fig. 2. There was high collinearity between these awareness items, so we created a composite measure of awareness for the multivariable analyses. Participants who had not previously heard of HPV or the vaccination programmes for girls or boys scored 0, those who had heard of HPV but not the vaccines scored 1, those who had heard of the vaccine programmes for girls and boys but were not aware of HPV, or who were aware of HPV and one (but not both) of the vaccines scored 2, and those who had heard of HPV, and the vaccines for girls and boys scored 3.

Attitudes to the HPV vaccine: We included nine items assessing attitudes to the HPV vaccine with responses on 5 point Likert scales (see Q8 in https://osf.io/yk2xh/ for the exact wording). These items were developed with reference to the previous literature [15], [17], [22], [23], [24] to include the most commonly reported barriers to HPV vaccination (lack of information, concern about novelty and side-effects, and worry that the vaccine might have an impact on sexual behaviour), social norms, perceived efficacy of the vaccine, perceptions of the health impact of HPV and the child’s future risk, and generally negative attitudes to vaccination. Attitudes were assessed after information exposure.

Descriptive variables: We assessed participants’ socio-demographic characteristics (see Table 1), age and sex of the index child, the participants’ role in vaccine decision-making for their children and previous refusal or delay of a vaccine for any child.

**Table 1:** Weighted sample characteristics (n = 1156) by sex of index child.

Participant characteristics	N (%)		
	All N = 1156	Boy index child N = 590	Girl index child N = 566
*Participant age* (range: 25–72) (Mean; SE)	40.5 (0.21)	40.5 (0.29)	40.5 (0.30)

*Participant sex*			
Male	451 (39.0)	239 (40.5)	211 (37.4)
Female	705 (61.0)	351 (59.5)	354 (62.6)

*Social grade*			
AB (high)	256 (22.2)	117 (19.8)	140 (24.7)
C1	339 (29.4)	177 (30.0)	163 (28.7)
C2	300 (25.9)	163 (27.6)	137 (24.2)
DE (low)	261 (22.6)	134 (22.7)	127 (22.4)

*Ethnic background*			
White (British/Irish/other)	923 (80.1)	471 (80.2)	452 (80.0)
Non-White	229 (19.9)	116 (19.8)	113 (20.0)

*Marital status*			
Married/Cohabiting	919 (79.5)	473 (80.2)	446 (78.7)
Single	139 (12.1)	71 (12.0)	69 (12.1)
Divorced/Separated/Widowed	98 (8.5)	46 (7.8)	52 (9.1)
*Index child’s age* (range: 8–13) (Mean; SE)	10.5 (0.04)	10.4 (0.05)	10.6 (0.05)

*Index child’s school year*			
Year 5 (age 9–10 years)*	355 (30.7)	190 (32.3)	164 (29.0)
Year 6 (age 10–11 years)	394 (34.1)	197 (33.4)	197 (34.8)
Year 7 (age 11–12 years)	407 (35.2)	202 (34.3)	205 (36.2)

*Ever refused a vaccine for a child?*			
No, never refused	1060 (91.7)	535 (90.8)	524 (92.7)
Yes, have previously refused	63 (5.4)	35 (6.0)	27 (4.8)
Missing/unsure	34 (2.8)	19 (3.2)	14 (2.5)

*Role in vaccine decision-making for children*			
Mainly my decision	453 (39.3)	231 (39.4)	222 (39.3)
Mainly my partner’s decision	121 (10.5)	71 (12.1)	49 (8.8)
We decide together	562 (48.8)	278 (47.5)	283 (50.1)
Other	17 (1.4)	6 (1.0)	11 (1.9)

Unweighted n = 1049; N may vary slightly between variables due to rounding of weighted n and missing data (max. n = 4 with missing data for any variable) *Some children were slightly outside these age ranges due to being a school year higher or lower than would be expected for their age. SE = Standard error.

### 2.5 Analysis

Weights calculated by Kantar TNS to adjust for non-response bias were applied to the data. All analyses were carried out in SPSS v25 using the ‘complex samples’ function to apply the weighting variable. Between-group differences in proportions were calculated using the complex samples chi-square procedure which calculates an adjusted F statistic to take account of weighting. Significance is based on the adjusted F and its degrees of freedom. We used multinomial logistic regression to explore predictors of having decided to/not to allow the index child to be vaccinated compared with being undecided, adjusting for child’s age and parent’s ethnic group and adding awareness and attitudes into the model in a stepwise manner.

The HPV vaccine attitude items were recoded so that a higher score indicated more negative attitudes. Cronbach’s alpha for combining all items (excluding ‘I believe that HPV infection could have serious health consequences for my child’ which had a low item-to-total correlation and reduced the alpha) was acceptable (0.78) so a mean score was created across the other 8 items. A prorated score was calculated for all participants who completed at least 5 out of 8 items. The mean scale score was 2.69 (standard error 0.02) (where range is 1–5 and low score indicates positive attitude). The scores were divided into quartiles for analysis, to allow for easier interpretation of odds ratios.

## 3 Results

### 3.1 Sample

Of 11,521 people aged 25 and over who took part in the omnibus survey and were assessed for eligibility, 1056 met the inclusion criterion of having a child in school years 5, 6 or 7. We excluded 7 participants who answered fewer than half of the questions, leaving a sample of 1049 for analysis (weighted n = 1156). Demographic characteristics of the sample are shown in Table 1 and unweighted data are presented alongside weighted data in Appendix 1.

Participants had a mean age of 40 years, 61% were mothers and there was a good distribution across occupational social class grades. Overall, 80% were from a white ethnic background and 80% were married or cohabiting. The index children had a mean age of just over 10 years and were evenly distributed by school year and sex. In relation to previous vaccination behaviour, 92% of participants reported never having refused a vaccine for any of their children, around half said vaccine decision-making was done jointly with their partner, and just under 40% reported being the main vaccine decision-maker for their child(ren).

### 3.2 HPV awareness

Overall 55% of participants had heard of HPV. The same proportion had heard about HPV vaccination for girls (55%) but fewer were aware that the vaccine would soon be offered to boys (23%). There were no differences in awareness between parents whose index child was a boy and those whose index child was a girl (see Table 2).

**Table 2:** Parents’ PAPM stage for HPV vaccination, HPV and vaccine awareness and vaccine attitudes by sex of index child.

	All N = 1156 N (%)	Sex of index child		Between group sex difference Adjusted F (df), p
		Boy N = 590 N (%)	Girl N = 566 N (%)	
*PAPM stage for HPV vaccination*				
Decided to vaccinate (Stage 5)	718 (62.1)	330 (55.9)	388 (68.6)	8.59 (2,2092), p < .0001*
Undecided (Stage 3)	323 (27.9)	194 (32.9)	129 (22.7)	
Decided not to vaccinate (Stage 4)	115 (10.0)	66 (11.1)	49 (8.7)	

*Heard of HPV before?*				
Yes	639 (55.3)	313 (53.0)	327 (57.8)	2.25 (1,1048), p = .13
No/Not sure	516 (44.7)	227 (47.0)	239 (42.2)	

*Heard of HPV vaccine for girls?*				
Yes	638 (55.2)	313 (53.1)	325 (57.5)	1.84 (1,1048), p = .18
No/Not sure	517 (44.8)	276 (46.9)	240 (42.5)	

*Heard HPV vaccine will be offered to boys?*				
Yes	267 (23.1)	132 (22.4)	135 (24.0)	0.33 (1,1048), p = 0.57
No/Not sure	888 (76.9)	458 (77.6)	430 (76.0)	

*Vaccine attitudes (% agree/strongly agree)*				
Need more information	621 (53.9)	352 (60.0)	269 (47.6)	14.63 (1,1048), p < .0001*
Concern about possible side-effects	379 (33.0)	205 (34.9)	174 (31.0)	1.64 (1,1048), p = .20
The HPV vaccine is too new	319 (27.8)	180 (30.8)	139 (24.7)	4.63 (1,1048), p = .03
HPV could have serious health consequences	442 (38.6)	216 (37.1)	225 (40.2)	0.93 (1,1048), p = .34
I don’t agree with vaccines	165 (14.3)	84 (14.3)	81 (14.4)	0.001 (1,1048), p = .98
My child may one day be at risk of HPV	464 (40.7)	215 (36.8)	249 (44.8)	6.14 (1,1048), p = .01
My child’s other parent would want us to vaccinate	621 (54.2)	284 (48.7)	336 (59.9)	12.03 (1,1048), p = .001*
HPV vaccine is effective	454 (39.7)	199 (34.2)	255 (45.4)	12.31 (1,1048), p < .0001*
HPV vaccine might make my child more likely to have sex	64 (5.8)	41 (7.4)	22 (4.2)	4.49 (1,1048), p = .03

Applying a Bonferroni adjustment for multiple testing (13 variables) gave us a critical p-value of 0.004.

* indicates statistical significance at the Bonferroni adjustment critical p-value 0.004.

### 3.3 PAPM stage for HPV vaccination

Following exposure to brief information about HPV, the vaccine and the imminent extension of the school-based programme to include boys (Box 1), parents of girls and boys were asked whether they would consent to the vaccine for their index child. Overall, 62% said that they would allow their child to be vaccinated, 28% were undecided and 10% said they would not. As shown in Table 2, there were significant differences in PAPM stage between parents of boys and girls, with parents of girls being more likely to have decided to vaccinate (69% compared with 56%) and parents of boys more likely to be undecided (33% vs. 23%). The proportion who would not allow their child to be vaccinated was similar (11% for parents of boys vs. 9% for parents of girls).

### 3.4 Attitudes to vaccinations

The proportion of participants agreeing or strongly agreeing with each of the attitude statements is shown in Table 2. Overall, 54% needed more information to make their decision, 33% were concerned about side-effects of the HPV vaccine and 28% agreed that the HPV vaccine was ‘too new’. Around 40% believed: that HPV could have serious health consequences (39%); that their child may one day be at risk of infection (41%); and that the vaccine was effective at preventing HPV (40%). Over half thought that their child’s other parent would want them to be vaccinated. Only a very small proportion (6%) believed that the vaccine might make their child more likely to have sex, and 14% said that they did not agree with vaccines in general. Parents whose index child was a girl were significantly more likely to agree that their child’s other parent would want to vaccinate and that the HPV vaccine is effective. Parents of boys were more likely to agree that they needed more information.

### 3.5 Predictors of PAPM stage for HPV vaccination

Having established that PAPM stage for HPV vaccination varied by child’s sex (see Table 2) we then explored other predictors of stage (see Table 3). There was no association between child’s school year and parent’s PAPM stage, but parent’s sex, awareness of HPV and the vaccines, previous vaccine refusal and all the attitude items showed significant associations with stage.

**Table 3:** Demographic, awareness and attitude differences by PAPM stage.

Variables	Stage 3 (Undecided) N = 323	Stage 4 (Decided not to vaccinate) N = 115	Stage 5 (Decided to vaccinate) N = 718	Between group difference Adjusted F (df), p
	n (row %)	n (row %)	n (row %)	
*All*	323 (27.9)	115 (10.0)	718 (62.1)	
*Parent’s sex*				
Male	161 (35.6)	45 (10.0)	245 (54.4)	20.8 (2,2093)
Female	162 (23.0)	70 (9.9)	473 (67.1)	p < .0001*

*School year of the child*				
Year 5	89 (25.2)	40 (11.2)	226 (63.7)	2.1 (4,4184)
Year 6	116 (29.4)	37 (9.3)	242 (61.4)	p = 0.75
Year 7	118 (28.9)	39 (9.6)	251 (61.5)	

*Heard of HPV before?*				
Yes	123 (19.2)	52 (8.2)	464 (72.6)	62.1 (2,2093)
Not sure/no	199 (38.6)	63 (12.2)	254 (49.2)	p < .0001*

*Heard of HPV vaccine for girls?*				
Yes	113 (17.7)	51 (8.0)	475 (74.4)	84.5 (2,2093)
Not sure/no	209 (40.4)	64 (12.4)	244 (47.1)	p < .0001*

*Heard HPV vaccine will be offered to boys?*				
Yes	32 (12.1)	17 (6.5)	218 (81.4)	50.9 (2,2096)
Not sure/no	290 (32.6)	98 (11.0)	500 (56.4)	p < .0001*

*Ever refused a vaccine?*				
No	286 (27.0)	82 (7.8)	691 (65.2)	71.4 (2,2092)
Yes	18 (29.3)	26 (40.8)	19 (29.9)	p < .0001*

*Attitude items (agree/strongly agree)*				
Need more information	262 (42.2)	53 (8.5)	306 (49.3)	60.2 (2,2095), p < .0001*
Concern about possible side-effects	125 (32.9)	59 (15.6)	195 (51.5)	14.5 (2,2093), p < .0001*
The HPV vaccine is too new	129 (40.6)	51 (16.0)	139 (43.5)	28.9 (2,2095), p < .0001*
HPV could have serious health consequences	63 (14.2)	34 (7.7)	345 (78.0)	34.0 (2,2095), p < .0001*
I don’t agree with vaccines	49 (29.5)	45 (27.5)	71 (43.0)	31.0 (2,2096), p < .0001*
My child may one day be at risk of HPV	66 (14.3)	26 (5.6)	372 (80.1)	44.0 (2,2096), p < .0001*
My child’s other parent would want us to vaccinate	62 (10.0)	28 (4.5)	531 (85.5)	138.6 (2,2095), p < .0001*
HPV vaccine is effective	35 (7.8)	16 (3.5)	403 (88.7)	98.8 (2,2095), p < .0001*
HPV vaccine might make my child more likely to have sex	14 (22.8)	14 (21.9)	35 (55.3)	4.8 (2,2095), p = .009

Significance testing uses a critical p-value of 0.003 to adjust for multiple comparisons.

For attitude items, 2x3 chi-square tests were used to compare agreement/strong agreement vs. other response across the 3 PAPM stages.

* Indicates p < .003.

The proportion of parents who had decided not to vaccinate was relatively consistent across sex, awareness and attitude sub-groups, at around 8–15%. One notable exception was that among parents who had previously refused a vaccine for a child, this figure rose to 40%. In addition, over 20% of parents who did not agree with vaccines in general and who believed the HPV vaccine might make their child more likely to have sex had decided not to vaccinate.

There was greater variation in the proportion of parents who had decided to vaccinate or who were undecided by parental sex, HPV awareness and vaccine attitudes. Parents who had heard of HPV, the vaccine for girls and plans to vaccinate boys were more likely to have decided to vaccinate (73%, 74% and 81% willing respectively, compared with 62% overall). Parents who needed more information, were concerned about side-effects or who thought the vaccine was too new were more likely to be undecided about vaccination (42%, 33% and 41% undecided compared with 28% overall). Most parents who agreed that the vaccine is effective (89%), that their child’s other parent would want to vaccinate (86%), that their child may one day be at risk of HPV (80%) and that HPV could have serious health consequences (78%) were at the ‘decided to vaccinate’ stage.

We re-ran these analyses stratifying by sex and found that patterns of association between predictors and PAPM stage were similar for parents of boys and girls (see Appendix 2). The exceptions were that parents’ gender, previous vaccine refusal and not agreeing with vaccines in general was more strongly associated with PAPM stage for parents of boys, whereas awareness of HPV and the girls’ vaccine, and the belief that the vaccine might make one’s child more likely to have sex were more strongly associated with stage when the index child was a girl.

### 3.6 Multivariable analyses

Finally, we carried out a series of multinomial logistic regression analyses exploring predictors of deciding not to or deciding to allow one’s child to be vaccinated, compared with being undecided (see Table 4). Model 1, with a pseudo R^2^ of 12%, included sex (of parent and child) and past vaccine refusal. Model 2, which added HPV and vaccine awareness, had a pseudo R-square of 21%, and Model 3, which also included vaccine attitudes, had a pseudo R^2^ of 47%. All analyses were adjusted for child’s age and parent’s ethnic group (white British vs. other).

**Table 4:** Multinomial logistic regression showing predictors of having decided to (or not to) vaccinate against HPV (compared with being undecided) Weighted n = 1,115 (Models 1 and 2) and 1,113 (Model 3).

	Odds of having decided to vaccinate (compared with being undecided)			Odds of having decided not to vaccinate (compared with being undecided)		
	Model 1	Model 2	Model 3	Model 1	Model 2	Model 3
Nagelkerke (pseudo R^2^)	0.12	0.21	0.47	0.12	0.21	0.47
*Parent’s sex*						
Male	Ref	Ref	Ref	Ref	Ref	Ref
Female	**1.88 (1.37**–**2.56)**	**1.43 (1.03**–**2.00)**	**1.62 (1.10**–**2.39)**	1.52 (0.92–2.52)	1.55 (0.91–2.61)	1.47 (0.87–2.47)

*Index child’s sex*						
Boy	Ref	Ref	Ref	Ref	Ref	Ref
Girl	**1.80 (1.32**–**2.45)**	**1.77 (1.29**–**2.44)**	**1.52 (1.05**–**2.20)**	1.20 (0.73–1.98)	1.19 (0.73–1.95)	1.21 (0.74–1.98)

*Past vaccine refusal*						
Never	Ref	Ref	Ref	Ref	Ref	Ref
Ever	**0.39 (0.19**–**0.81)**	**0.31 (0.14**–**0.68)**	0.48 (0.22–1.09)	**4.96 (2.41**–**10.21)**	**4.70 (2.31**–**9.57)**	**4.77 (2.26**–**10.05)**

HPV/vaccine awareness*						
0 (None)		Ref	Ref		Ref	Ref
1		1.22 (0.68–2.18)	1.34 (0.72–2.50)		0.62 (0.21–1.80)	0.67 (0.24–1.87)
2		**2.44 (1.66**–**3.56)**	1.19 (0.76–1.86)		0.86 (0.47–1.60)	0.81 (0.44–1.49)
3 (All)		**7.73 (4.48**–**13.35**)	**3.39 (1.74**–**6.58)**		**2.15 (1.06**–**4.39)**	1.67 (0.80–3.49)

*Vaccine attitudes (quartiles)*						
1 (most positive)			**60.96 (24.49**–**151.76)**			1.97 (0.52–7.43)
2			**12.65 (7.72**–**20.72)**			1.10 (0.52–2.30)
3			**1.70 (1.07**–**2.70)**			0.80 (0.46–1.40)
4 (most negative)			Ref			Ref

* 0 = not aware of HPV or either vaccine; 1 = aware of HPV but neither vaccine; 2 = aware of at least one vaccine (for girls or boys) but not HPV, or HPV and one vaccine; 3 = aware of HPV and vaccines for girls and boys. All analyses are adjusted for child’s age and parent’s ethnic group.

*Parent’s sex* was significantly associated with having decided to vaccinate in all three models, with mothers more likely to have decided to vaccinate than fathers. There was no association between parent’s sex and having decided not to vaccinate. The pattern was similar for *child’s sex*, with parents of girls more likely to have decided to vaccinate than parents of boys, but no association with deciding not to vaccinate.

*Past refusal of a vaccine* was associated with lower odds of deciding to vaccinate in the first two models, but the association was no longer significant once attitudes were added to the model (Model 3). There was a consistent association between past refusal and having decided not to vaccinate across all three models (OR: 4.96, 4.70 and 4.77 for Models 1, 2 and 3 respectively).

*HPV and vaccine awareness* showed a dose-response association with having decided to vaccinate in Model 2 (with higher awareness associated with greater odds of deciding to vaccinate), but the effect was largely attenuated when attitudes were added to the model (Model 3). Being aware of HPV and both vaccines was also significantly predictive of deciding not to vaccinate in Model 2, but this association was no longer significant in Model 3.

*HPV vaccine attitudes* were by far the strongest predictor of having decided to vaccinate, with massively increased odds in the quartile with the most positive attitudes (OR: 60.96, 95% confidence interval: 24.49–151.76). No such association was seen for deciding not to vaccinate, suggesting that overall attitudes were similar in the ‘decided not’ and ‘undecided’ groups.

## 4 Discussion

This study is the first to explore awareness, attitudes and intentions in relation to HPV vaccination among parents of both boys and girls in England and Wales. Despite low awareness of the imminent introduction of HPV vaccination for boys, the findings are broadly reassuring and suggest that gender-neutral vaccination will be well-received. As would be expected, awareness of the plan to offer the vaccine to boys was lower than awareness of the existing programme for girls (23% compared with 55%), and parents of boys were more likely than parents of girls to be undecided about whether they would vaccinate (33% vs. 23%). The most-endorsed barrier to vaccination among all parents was needing more information. Mothers were more likely to have decided to vaccinate than fathers and those who had heard of HPV and the vaccine programmes (for girls and boys) were also more likely to say they would vaccinate. However, positive attitudes seemed to have the greatest influence on the decision to vaccinate. Around 10% of parents said they would not allow their child to have the vaccine, with no difference by child or parents’ sex. The only significant predictor of having decided not to vaccinate in the fully adjusted model was previous vaccine refusal.

The factors we found to be predictive of having decided to vaccinate are similar to those identified in a recent systematic review and *meta*-analysis [15] which found HPV vaccine uptake behaviour was associated with safety concerns, belief in vaccines in general, perceived benefits of the HPV vaccine and HPV vaccine awareness, with uptake higher for girls than boys, and in mothers than fathers. The review did not include any studies carried out in the UK and although our findings relate to intention rather than vaccine uptake, they suggest that the pattern of associations is likely to be similar in the UK. The review also identified physician recommendation as a key predictor of HPV vaccine uptake. In the UK, where the vaccine is offered through schools, there is little opportunity for a discussion with a health professional before the decision is made, but it may be useful for health professionals (e.g. in primary care) to be alert to opportunities to mention the vaccine to eligible young people and their parents.

In terms of awareness of HPV and the vaccine, our findings are strikingly similar to an earlier smaller-scale UK-based study [14], suggesting that awareness has not increased appreciably in the ~2 years between the studies. Although the girls’ vaccination programme and the inclusion of HPV testing within the cervical screening programme have led to increases in HPV knowledge and awareness over the last decade, it is concerning that almost half of our sample had not heard of the virus, so ongoing efforts are needed to facilitate informed participation in the vaccination and screening programmes. A recent *meta*-ethnography analysis found that poor knowledge and low awareness was associated with lower acceptability of HPV vaccination in parents of boys, although other levels of influence (interpersonal, community and systemic) were also important [25].

A novel aspect of our study was the use of the PAPM as a framework within which to categorise parents’ stage of vaccine uptake decision-making in England and Wales. Like Shapiro et al [17] in Canada, we found that parents of boys were more likely to be undecided about vaccinating, compared with parents of girls, but the proportion who had decided against vaccination was independent of the sex of the child (and was small at ~ 10% in both studies). This is encouraging in light of evidence that being undecided reflects a ‘flexible hesitancy’ that is amenable to change over time [19]. Identifying this group of uncertain parents who are in need of more information provides useful insight to inform interventions. Parents of children being offered the vaccine in England from September 2019 will be provided with a gender-neutral NHS leaflet (https://assets.publishing.service.gov.uk/government/uploads/system/uploads/attachment_data/file/812484/PHE_HPV_vaccination_leaflet.pdf) which explains the rationale for extending the programme to boys, and signposts to more information on safety and efficacy available online. Our findings support the need for provision of information, especially to address concerns about the safety and novelty of the vaccine.

Barriers among parents who were unwilling to vaccinate may be harder to address as they seem to relate to broader and more stable anti-vaccination attitudes [19], as evidenced by the strong association in our study with previous vaccine refusal. Although there is increasing concern about vaccine hesitancy, in the World Health Organisation and elsewhere [26], the ‘anti-vaxx’ group in our study appeared to be small. Although our findings are broadly reassuring, the potential for an anti-vaccination minority to have a sudden and dramatic effect on uptake of HPV vaccination at a national level should not be underestimated (e.g. [27]). It is essential that public health bodies are prepared to respond swiftly and appropriately to restore vaccine confidence in such circumstances, as was done successfully following a sharp decline in HPV vaccine uptake in Ireland [28].

### 4.1 Strengths and limitations

The study benefited from a large sample that was broadly representative of the general population. The survey was nested within a general market research ‘omnibus’, which reduces the chances that the sample was self-selected in relation to vaccine attitudes. A limitation is that Kantar TNS do not collect any information on participation rates or details of non-responders to the overall survey, so we are not able to calculate a response rate. Our sample was biased towards mothers (61% of responders were women), but this likely reflects the fact that mothers tend to be the primary caregivers [29]. We were not able to include questions about older children so we do not know if participants had experience of the HPV vaccine for an older child.^1^ Finally, the findings are hypothetical, assessing intention to vaccinate following provision of only very brief information, which did not explicitly emphasise the benefits of the vaccine for boys. Fuller information may lead to different decision-making. It is very likely that a significant (but unknown) proportion of parents who were ‘undecided’ will choose to vaccinate their children once they have been given more comprehensive information. In addition, although intention is a strong predictor of behaviour, including in the HPV vaccination context [15], some parents with positive intentions may not vaccinate their children due to practical rather than attitudinal barriers. There was a discrepancy between the proportion of parents who said they did not agree with vaccines in general (14% endorsed the statement) and those who reported ever having refused a vaccine for a child (5%), which suggests some parents may vaccinate despite negative attitudes.

### 4.2 Implications

Our findings suggest that raising awareness of HPV and the vaccination programme (particularly for boys) will be an essential first step towards increasing the proportion of parents who are willing to vaccinate. Information materials for parents should be clear about vaccine safety and should emphasise the large numbers of children who have been vaccinated to address concerns about the novelty of the vaccine and its possible side-effects. Although there were some differences in attitudes to HPV vaccination between parents of girls and boys, for the most part our work suggests parents of boys and girls have similar attitudes toward HPV vaccination and so providing gender-specific information is not likely to be necessary. Rather, gender-neutral information that addresses the main concerns identified by all parents is likely to be an acceptable approach. However, this information should convey the messages that boys are likely to be at risk of getting HPV and that the vaccine has been shown to be effective in boys.

### 4.3 Conclusions

Our findings suggest broad acceptability of HPV vaccination for boys in England and Wales, with few parents saying that they would not vaccinate their sons. However, awareness of HPV and the vaccine, and particularly plans to vaccinate boys, remains low and a significant proportion of parents of both boys and girls said they needed more information to make the decision. Public awareness campaigns and provision of information addressing key questions and concerns to parents of children being offered the vaccine will be important to ensure good HPV vaccine coverage in boys and girls.

## Declaration of Competing Interest

The authors declare that they have no known competing financial interests or personal relationships that could have appeared to influence the work reported in this paper.

## Appendix A Supplementary material

Supplementary data 1

Appendix 1 Sample characteristics (unweighted).

Appendix 2 PAPM stage by demographic and knowledge sub-group; stratified by child’s gender (N = 1156 (WEIGHTED).

## Footnotes

1 We did ask a single question about vaccination of an older daughter but the distribution of responses suggested the question was misinterpreted (there was low apparent uptake of the HPV vaccine for cohorts in which coverage is known to have been high). We therefore decided not to report these responses or include this variable in analyses due to the unknown validity of the data.
