# Supplementary material for: Decision-making about HPV vaccination in parents of boys and girls: A population-based survey in England and Wales
Source: Vaccine. 2020 Jan 29;38(5):1040–7. doi: 10.1016/j.vaccine.2019.11.046 (PMC6996150; doi:10.1016/j.vaccine.2019.11.046)
Supplement: Appendix 1 — Sample characteristics (unweighted). [file mmc2.docx]

Appendix 1

Sample characteristics (unweighted)

| **Participant characteristics** | ***N (%)*** | |
| --- | --- | --- |
|  | ***All (weighted)***  ***N=1,156*** | ***All (unweighted)***  ***N=1,049*** |
|  |  |  |
| Participant age (range: 25-72) (Mean; SE) | 40.5 (0.21) | 39.7 (0.22) |
|  |  |  |
| Participant sex |  |  |
| Male | 451 (39.0) | 370 (35.3) |
| Female | 705 (61.0) | 679 (64.7) |
|  |  |  |
| Social grade |  |  |
| AB (high) | 256 (22.2) | 176 (16.8) |
| C1 | 339 (29.4) | 236 (22.5) |
| C2 | 300 (25.9) | 298 (28.4) |
| DE (low) | 261 (22.6) | 339 (32.3) |
|  |  |  |
| Ethnic background |  |  |
| White (British/Irish/other) | 923 (80.1) | 832 (79.3) |
| Non-White | 229 (19.9) | 213 (20.3) |
|  |  |  |
| Marital status |  |  |
| Married/Cohabiting | 919 (79.5) | 805 (76.7) |
| Single | 139 (12.1) | 157 (15.0) |
| Divorced/Separated/Widowed | 98 (8.5) | 87 (8.3) |
|  |  |  |
| Index child’s age (range: 8-13) (Mean; SD) | 10.5 (0.04) | 10.5 (0.03) |
|  |  |  |
| Sex of index child |  |  |
| Female | 566 (49.0) | 510 (48.6) |
| Male | 590 (51.0) | 539 (51.4) |
|  |  |  |
| Index child’s school year |  |  |
| Year 5 | 355 (30.7) | 325 (310) |
| Year 6 | 394 (34.1) | 355 (33.8) |
| Year 7 | 407 (35.2) | 369 (35/2) |
|  |  |  |
| Ever refused a vaccine for a child? |  |  |
| No, never refused | 1060 (91.7) | 958 (91.3) |
| Yes, have previously refused | 63 (5.4) | 56 (5.3) |
| Missing/unsure | 34 (2.8) | 35 (3.3) |
|  |  |  |
| Vaccine decision-making for children |  |  |
| Mainly my decision | 453 (39.3) | 450 (42.9) |
| Mainly my partner’s decision | 212 (10.5) | 109 (10.4) |
| We decide together | 562 (48.8) | 468 (44.6) |
| Other | 17 (1.4) | 18 (1.7) |
|  |  |  |
| Heard of HPV before? |  |  |
| Yes | 639 (55.3) | 559 (53.3) |
| No/Not sure | 516 (44.7) | 489 (46.6) |
|  |  |  |
| Heard of HPV vaccine for girls |  |  |
| Yes | 638 (55.2) | 561 (53.5) |
| No/Not sure | 517 (44.8) | 487 (46.4) |
|  |  |  |
| Heard that HPV vaccine will be offered to boys |  |  |
| Yes | 267 (23.1) | 231 (22.0) |
| No/Not sure | 888 (76.9) | 817 (77.9) |
|  |  |  |
| PAPM stage for HPV vaccine decision |  |  |
| Stage 5: Decided to act (‘Yes’) | 718 (62.1) | 639 (60.9) |
| Stage 3 : Undecided (‘I’m not sure yet’) | 323 (27.9) | 294 (28.0) |
| Stage 4: Decided no (’No’) | 115 (10.0) | 116 (11.1) |
