# Supplementary material for: Decision-making about HPV vaccination in parents of boys and girls: A population-based survey in England and Wales
Source: Vaccine. 2020 Jan 29;38(5):1040–7. doi: 10.1016/j.vaccine.2019.11.046 (PMC6996150; doi:10.1016/j.vaccine.2019.11.046)
Supplement: Appendix 2 — PAPM stage by demographic and knowledge sub-group; stratified by child’s gender (N = 1156 (WEIGHTED). [file mmc3.docx]

| *Appendix 2: PAPM stage by demographic and knowledge sub-group; stratified by child’s gender (N=1,156 (WEIGHTED)* | | | | | | | | |
| --- | --- | --- | --- | --- | --- | --- | --- | --- |
|  | **Index child is a boy** | | | | **Index chid is a girl** | | | |
| **Variables** | **Undecided** | **Decided not to vaccinate** | **Decided to vaccinate** | **Between group difference Adjusted F (df), p** | **Undecided** | **Decided not to vaccinate** | **Decided to vaccinate** | **Between group difference Adjusted F (df), p** |
|  | n (row %) | n (row %) | n (row %) |  | n (row %) | n (row %) | n (row %) |  |
| *All* | 100 (41.8%) | 66 (11.1%) | 330 (55.9%) |  | 129 (22.7%) | 49 (8.7%) | 388 (68.6%) |  |
|  |  |  |  |  |  |  |  |  |
| *Parent’s gender* |  |  |  | 6.1 (2,1074), p=.002 |  |  |  | 3.3 (2,1016), p=.04 |
| Male | 100 (41.8%) | 25 (10.6%) | 114 (47.6%) |  | 61 (28.7%) | 20 (9.4%) | 131 (62.0%) |  |
| Female | 94 (26.9%) | 40 (11.5%) | 216 (61.6%) |  | 68 (19.1%) | 30 (8.3%) | 257 (72.5%) |  |
|  |  |  |  |  |  |  |  |  |
| School year |  |  |  | 0.8 (4,2149), p=0.52 |  |  |  | 0.9 (4,2029), p=.47 |
| Year 5 (age 9-10 years) | 55 (28.8%) | 22 (11.8%) | 113 (59.4%) |  | 35 (21.0%) | 17 (10.5%) | 113 (68.5%) |  |
| Year 6 (age 10-11 years) | 63 (31.8%) | 23 (11.6%) | 112 (56.6%) |  | 53 (26.9%) | 14 (7.0%) | 130 (66.1%) |  |
| Year 7 (age 11-12 years) | 77 (37.9%) | 21 (10.2%) | 105 (52.0%) |  | 41 (20.0%) | 18 (9.0%) | 145 (71.0%) |  |
|  |  |  |  |  |  |  |  |  |
| *Awareness of HPV* |  |  |  | 6.7 (2,1075) , p=.001 |  |  |  | 26.3 (2,1016), p<.0001 |
| Yes | 83 (26.5%) | 31 (10.0%) | 199 (63.5%) |  | 40 (12.2%) | 21 (6.4%) | 266 (81.3%) |  |
| Not sure/no | 111 (40.1%) | 35 (12.5%) | 131 (47.4%) |  | 88 (36.8%) | 28 (11.9%) | 122 (51.3%) |  |
|  |  |  |  |  |  |  |  |  |
| *Awareness of girls’ vaccine* |  |  |  | 12.7 (2,1075), p<.0001 |  |  |  | 30.2 (2,1013), p<.0001 |
| Yes | 74 (23.5%) | 33 (10.6%) | 206 (65.9%) |  | 39 (12.0%) | 18 (5.4%) | 268 (82.5%) |  |
| Not sure/no | 121 (43.6%) | 33 (11.8%) | 123 (44.6% |  | 89 (36.9%) | 32 (13.2%) | 120 (49.9%) |  |
|  |  |  |  |  |  |  |  |  |
| *Awareness of boys’ vaccine* |  |  |  | 13.6 (2,1076), p<.0001 |  |  |  | 10.7 (2,1018), p<.0001 |
| Yes | 20 (15.5%) | 9 (6.7%) | 103 (77.8%) |  | 12 (8.7%) | 9 (6.3%) | 115 (85.0%) |  |
| Not sure/no | 174 (37.9%) | 57 (12.4%) | 227 (49.6%) |  | 116 (27.0%) | 41 (9.5%) | 273 (63.5%) |  |
|  |  |  |  |  |  |  |  |  |
| *Ever refused a vaccine for a child?* |  |  |  | 33.1 (2,1076), p<.0001 |  |  |  | 3.8 (2,1016), p=.02 |
| No | 173 (32.2%) | 42 (7.8%) | 321 (59.9%) |  | 114 (21.7%) | 40 (7.7%) | 370 (70.6%) |  |
| Yes | 12 (32.6%) | 19 (54.1%) | 5 (13.3%) |  | 7 (24.9%) | 6 (23.5%) | 14 (51.6%) |  |
|  |  |  |  |  |  |  |  |  |
| *Attitude items (agree/strongly agree)* |  |  |  |  |  |  |  |  |
| Need more information | 158 (45.0%) | 33 (9.4%) | 160 (45.5%) | 25.4 (2,1076), p<.0001 | 104 (38.4%) | 20 (7.3%) | 146 (54.2%) | 32.2 (2,1018), p<.0001 |
| Concern about possible side-effects | 73 (35.8%) | 36 (17.7%) | 95 (46.5%) | 7.7 (2,1075), p<.0001 | 51 (29.5%) | 23 (13.1%) | 100 (57.4%) | 6.9 (2,1016), p=.001 |
| The HPV vaccine is too new | 80 (44.3%) | 32 (17.8%) | 68 (37.9%) | 15.4 (2,1075), p<.0001 | 50 (35.7%) | 19 (13.7%) | 70 (50.6%) | 12.2 (2,1017), p<.0001 |
| HPV could have serious health consequences | 36 (16.7%) | 21 (9.8%) | 159 (73.5%) | 19.4 (2,1076), p<.0001 | 27 (11.8%) | 13 (5.7%) | 186 (82.4%) | 14.5 (2,1017), p<.0001 |
| I don’t agree with vaccines | 32 (38.5%) | 28 (33.3%) | 24 (28.2%) | 25.2 (2,1075), p<.0001 | 16 (20.2%) | 17 (21.4%) | 47 (58.3%) | 8.6 (2,1017), p<.0001 |
| My child may one day be at risk of HPV | 39 (18.0%) | 14 (6.5%) | 162 (75.5%) | 20.6 (2,1076), p<.0001 | 27 (11.1%) | 12 (4.8%) | 209 (84.1%) | 21.7 (2,1018), p<.0001 |
| My child’s other parent would want us to vaccinate | 33 (11.5%) | 13 (4.6%) | 239 (83.9%) | 76.7 (2,1074), p<.0001 | 29 (8.7%) | 15 (4.4%) | 292 (86.9%) | 58.7 (2,1018), p<.0001 |
| HPV vaccine is effective | 18 (9.1%) | 6 (2.9%) | 175 (88.0%) | 54.5 (2,1072), p<.0001 | 17 (6.7%) | 10 (4.0%) | 228 (89.3%) | 41.7 (2,1017), p<.0001 |
| HPV vaccine might make my child more likely to have sex | 12 (29.0%) | 7 (17.1%) | 22 (54.0%) | 0.72 (2,1076), p=.49 | 3 (11.3%) | 7 (30.9%) | 13 (57.8%) | 7.2 (2,999), p=.001 |
| Significance testing uses a critical p-value of .003 to adjust for multiple comparisons.  ^*^Indicates p<.003  For attitude items, 2x3 chi-square tests were used to compare agreement/strong agreement vs. other response across the 3 PAPM stages. | | | | | | | | |
